# Postoperative NEOadjuvant TEMozolomide followed by chemoradiotherapy versus upfront chemoradiotherapy for glioblastoma multiforme (NEOTEM) trial: Interim results

**DOI:** 10.1093/noajnl/vdae195

**Published:** 2024-11-14

**Authors:** Azadeh Sharifian, Ali Kazemian, Mostafa Farzin, Nikan Amirkhani, Borna Farazmand, Soheil Naderi, Alireza Khalilian, Ahmad Pourrashidi, Ghazaleh Amjad, Kasra Kolahdouzan, Romina Abyaneh, Paola Anna Jablonska, Reza Ghalehtaki

**Affiliations:** Department of Radiation Oncology, Cancer Institute, IKHC, School of Medicine, Tehran University of Medical Sciences, Tehran, Iran; Radiation Oncology Research Center, Cancer Research Institute, IKHC, Tehran University of Medical Sciences, Tehran, Iran; Department of Radiation Oncology, Cancer Institute, IKHC, School of Medicine, Tehran University of Medical Sciences, Tehran, Iran; Brain and Spinal Cord Injury Research Center, Neuroscience Institute, Tehran University of Medical Sciences, Tehran, Iran; Department of Radiation Oncology, Cancer Institute, IKHC, School of Medicine, Tehran University of Medical Sciences, Tehran, Iran; School of Medicine, Tehran University of Medical Sciences, Tehran, Iran; Radiation Oncology Research Center, Cancer Research Institute, IKHC, Tehran University of Medical Sciences, Tehran, Iran; Department of Neurosurgery, Iran University of Medical Sciences, Tehran, Iran; Radiation Oncology Research Center, Cancer Research Institute, IKHC, Tehran University of Medical Sciences, Tehran, Iran; Department of Radiation Oncology, Cancer Institute, IKHC, School of Medicine, Tehran University of Medical Sciences, Tehran, Iran; Department of Neurosurgery, Sina Hospital, Tehran University of Medical Sciences, Tehran, Iran; UPMC Hillman Cancer Center, Radiology Department, University of Pittsburgh, Pittsburgh, Pennsylvania, USA; Radiation Oncology Research Center, Cancer Research Institute, IKHC, Tehran University of Medical Sciences, Tehran, Iran; Department of Radiation Oncology, Cancer Institute, IKHC, School of Medicine, Tehran University of Medical Sciences, Tehran, Iran; Radiation Oncology Research Center, Cancer Research Institute, IKHC, Tehran University of Medical Sciences, Tehran, Iran; Radiation Oncology Department, Hospital Universitario de Navarra, Pamplona, Spain; Radiation Oncology Research Center, Cancer Research Institute, IKHC, Tehran University of Medical Sciences, Tehran, Iran; Department of Radiation Oncology, Cancer Institute, IKHC, School of Medicine, Tehran University of Medical Sciences, Tehran, Iran

**Keywords:** chemoradiotherapy, glioblastoma, MGMT methylation, neoadjuvant temozolomide, progression-free survival

## Abstract

**Background:**

Glioblastoma multiforme (GBM) is an aggressive brain tumor with poor survival rates despite current treatments. The standard of care (SOC) includes surgery, followed by radiotherapy plus concurrent and adjuvant chemotherapy with temozolomide (TMZ). This phase II trial assessed the safety and efficacy of neoadjuvant TMZ (nTMZ) before and during chemoradiotherapy in newly diagnosed GBM patients.

**Methods:**

Newly diagnosed GBM patients who underwent maximal safe resection were randomized into 2 groups. One received nTMZ before standard chemoradiotherapy and adjuvant TMZ (intervention). The other received standard chemoradiotherapy followed by adjuvant TMZ (control). Primary endpoints were progression-free survival (PFS) at 6 and 12 months. Secondary endpoints included overall survival, radiological and clinical responses, and adverse events.

**Results:**

Of 35 patients, 16 were in the intervention group and 19 in the control group. Median PFS was 9 months (95% CI: 3.93–14.06) versus 3 months (95% confidence interval [CI]: 1.98–4.01) in the control and intervention groups (*P* = .737), with a high progression rate (73.4%) during nTMZ treatment. The 6-month PFS rates were 58% versus 25% (*P* = .042), and 12-month PFS rates were 26% versus 25% (*P* = .390) in the control and intervention groups, respectively. Patients with unmethylated O^6^-methylguanine-DNA methyltransferase (MGMT) and those with good performance status (PS) had significantly worse PFS with nTMZ.  However, those who underwent larger extent of resection exhibited significantly better PFS  with nTMZ. Adverse events were similar between groups.

**Conclusions:**

Neoadjuvant TMZ before SOC chemoradiotherapy did not improve outcomes for newly diagnosed GBM patients and is unsuitable for those with unmethylated MGMT and good PS. However, It may benefit patients with near or gross total resection. Further research is needed to refine GBM treatment strategies.

## Lay Summary

Glioblastoma is an aggressive brain cancer with limited treatment options. Standard treatment involves surgery, followed by radiation and chemotherapy with a drug called temozolomide (TMZ). The authors of this study wanted to determine whether giving even more TMZ chemotherapy before radiation would help patients live longer. To do this they conducted a clinical trial where 35 patients were randomly assigned to receive additional early chemotherapy or standard treatment. Their results showed that giving additional early chemotherapy did not change how long patients lived. Side effects were similar between groups.

**Key Points**

- Neoadjuvant temozolomide (nTMZ) did not improve overall survival or progression-free survival in glioblastoma.
- Patients with unmethylated O^6^-methylguanine-DNA methyltransferase and good performance status had worse outcomes with nTMZ. However, those who underwent larger extent of resection had significantly better PFS with nTMZ.
- Adverse events were similar across both treatment groups.

**Importance of the Study**

This phase II trial evaluates the impact of neoadjuvant temozolomide (nTMZ) in newly diagnosed glioblastoma (GBM) patients compared to standard chemoradiotherapy. Despite the rationale for nTMZ as an early intervention, our study reveals that it does not enhance progression-free survival. It may be less effective in patients with unmethylated O^6^-methylguanine-DNA methyltransferase, good performance status, or those undergoing incomplete resections. These findings challenge the potential benefits of nTMZ and highlight its unsuitability for certain patient subgroups. Future research should focus on optimizing GBM treatment strategies, possibly incorporating patient-specific factors to better tailor therapies.

Glioblastoma (GBM) is the most common and lethal primary brain tumor in adults, which accounts for 75% of high-grade glial brain tumors.^1^ Despite the implementation of various treatment modalities, GBM prognosis remains poor, with median overall survival (OS) and progression-free survival (PFS) of approximately 14 months and 6.9 months, respectively.^2^

The current standard of care (SOC) for the treatment of GBM is based on the landmark trial published in 2005 by Stupp et al. This study showed that the addition of concurrent and adjuvant temozolomide (TMZ) to postoperative radiotherapy (RT) increased the median OS from 12.1 to 14.6 months and the 2- and 5-year OS from 10.4% to 26.5% and from 0% to 10% respectively when compared to postoperative RT alone.^3^ These results established the standard treatment for GBM, consisting of debulking surgery followed by concomitant chemoradiotherapy (CRT) and adjuvant TMZ. Subsequent studies failed to obtain better outcomes, with the exception of the addition of tumor-treating fields (TTF) to TMZ maintenance after CRT in the EF-14 trial, which resulted in increased median OS from 16 to 20.9 months.^4^ Nevertheless, the EF-14 study presented some limitations, including an open-label setting, and other randomized trials failed to reproduce these findings.^5^ As a result, the use of TTF remains infrequent, also in advanced neuro-oncology programs in developed countries.^6^ Subsequently, other agents have been explored in phase 3 randomized controlled trials (RCT). The addition of nivolumab, an anti-PD1 (programmed cell death protein 1) drug, to RT did not improve the OS rates in unmethylated MGMT (um-MGMT) patients.^7^ Lomustine plus TMZ showed some efficacy in methylated-MGMT (m-MGMT) patients, though at the cost of higher toxicity, limiting its use to younger patients with good performance status (PS).^8,9^ Considering the available evidence, RT and TMZ remain the safest, most effective, and readily accessible options for the majority of GBM patients with both m-MGMT and um-MGMT.

The efficacy of TMZ treatment was first shown in 1999 by clinical trials in the treatment of recurrent glioma. In these studies, the administration of TMZ before, during, and after RT was proven to be effective in this setting.^10^ However, the role of TMZ as neoadjuvant therapy in *de novo* GBM has not yet been defined. There are many theoretical advantages of neoadjuvant TMZ (nTMZ) use before RT: (a) O^6^-alkylguanine DNA alkyl-transferase, also known as O^6^-methylguanine-DNA methyltransferase (MGMT), is a DNA repair enzyme that antagonizes the effects of alkylating drugs. When the *MGMT* gene promoter is methylated, which occurs in about 45% of GBM cases, a better response to alkylating agents is observed.^11^ *MGMT* status is the strongest predictor of survival among glioma patients receiving alkylating drugs.^12^ Protracted treatment with TMZ (at least 7 days) can decrease MGMT activity, hence increasing the efficacy of this drug.^9^ Therefore, nTMZ before RT may enhance its activity and effect during subsequent RT treatment, (b) based on previous reports, nTMZ may result in tumor shrinkage, which can improve neurological symptoms and reduce radiation treatment volume.^13–15^ This may increase the possibility of delivering full-dose RT and lessen the side effects.^16^ Tumors with *MGMT* gene promoter methylation are more sensitive to chemotherapy. Therefore, if neoadjuvant chemotherapy can shrink the tumor size before radiation therapy, patients with MGMT-methylated tumors are likely to gain the most significant benefit from this approach, (c) it was shown that 53% of GBM patients present with tumor regrowth within 4 weeks after surgery.^11^ Currently, the routine interval between the surgery and the start of chemoradiotherapy lies in 4-5 weeks,^17^ and there is some evidence that starting RT earlier than 3 weeks is associated with worse outcomes.^18,19^ Also, in real-world settings, the interval between surgery and RT might exceed 6 weeks due to various socioeconomic factors.^19^ By using neoadjuvant chemotherapy, TMZ treatment can be started sooner than RT, which could theoretically reduce the risk of tumor regrowth in a subset of patients, and (d) performing MRI before RT helps in defining the radiation volume. However, surgery-induced reactive enhancements can appear immediately or within weeks following the tumor resection, affecting the delineation for radiation planning.^20^ This is particularly important considering the latest guidelines recommending narrower CTV (clinical target volume) margins than before.^21^ Starting TMZ after surgery and before RT, if proven effective, may eliminate the need to start RT sooner than 5 weeks. This may allow us to perform an MRI at a time when fewer artifacts are anticipated and provide the ideal time to obtain a more accurate RT plan.

Considering the poor outcomes despite the ongoing research and taking into account the potential benefit of neoadjuvant chemotherapy, we aimed to assess the feasibility and efficacy of adding nTMZ to chemoradiotherapy after surgery in patients with newly diagnosed GBM.

## Material and Methods

### Study Population

Patients with newly diagnosed, histologically confirmed GBM who underwent surgery, including different extent of resection such as biopsy, subtotal resection (STR), near-total resection (NTR), and gross tumor resection (GTR), were enrolled in this phase II trial. The inclusion criteria included: age 18–80 years, contrast-enhanced brain MRI with radiological evidence of high-grade glial tumor, pathological confirmation of GBM, Eastern Cooperative Oncology Group (ECOG) PS of 0–3, adequate hematopoietic (hemoglobin ≥ 9 g/dL, platelet count ≥ 100 000/µL, absolute neutrophil count [ANC] ≥ 1500/µL), renal (serum creatinine ≤ 1.5 mg/dL), and hepatic functions (aspartate transaminase [AST] and alanine transaminase [ALT] ≤ 3 times the upper limit of normal, bilirubin ≤ 1.5 mg/dL), and absence of severe systemic disease such as infection that requires injectable antibiotic treatment.

Patients with a history of prior or current evidence of other cancer diagnosis, prior radiation to the brain except for treatment of scalp squamous cell carcinoma (SCC) and/or basal cell carcinoma (BCC), low-grade or anaplastic glioma, limited PS, that is ECOG 2–3 due to reasons other than the brain tumor, pregnancy, breastfeeding, or any condition (medical, social, and psychiatric) that hinders the possibility of gathering information and follow-up were excluded from the study.

### Study Design and Treatment

This is a randomized phase II trial conducted at Iran Cancer Institute between 2020 and 2022. The study was approved by the Tehran University of Medical Sciences review board and ethics committee (code: IR.TUMS.IKHC.REC.1399.240), followed by registration in the Iranian repository of clinical trials (IRCT code #20150929024266N5).

The trial was designed to test the safety and efficacy of neoadjuvant chemotherapy with TMZ (before concomitant CRT and after surgery) followed by the SOC treatment (intervention arm) compared to the SOC treatment alone (control arm). Standard of care treatment was defined as maximal safe resection surgery followed by concomitant CRT and adjuvant chemotherapy with TMZ for at least 6 cycles, to a maximum of 12 cycles.

Patients with newly diagnosed GBM who had undergone maximal safe resection and had met all the inclusion criteria were randomized on a 1:1 basis. The trial statistician generated a randomization code using a computer-based random number table.

Patients in both arms were required to initiate the treatment within 3–5 weeks after the surgery. In the intervention arm, nTMZ was delivered for 3 cycles, followed by CRT and adjuvant TMZ for 3 cycles at least 4 weeks after RT completion. The TMZ was given at 150–200 mg/m^2^/d for 5 days (days 1–5) every 28 days in the neoadjuvant and adjuvant settings and at 75 mg/m^2^/d daily concurrently with RT. The RT schedule was 60 Gy in 30 sessions at 2 Gy per fraction, 5 days a week. If patients showed clinical progression during the neoadjuvant period, the RT prescription was adjusted to a hypofractionated regimen (40 Gy in 15 fractions) in limited PS.

**Figure 1.**
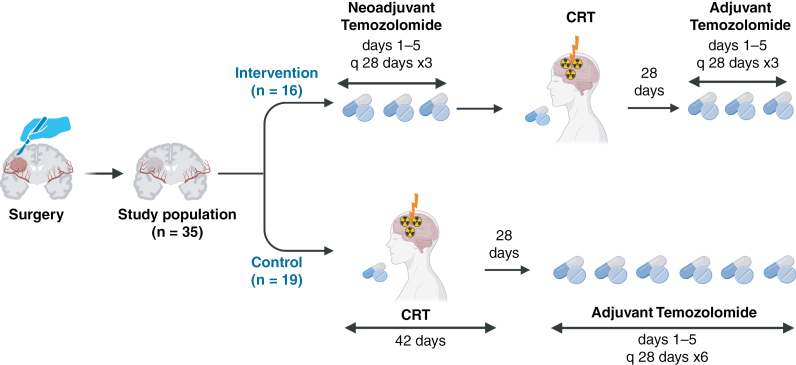
The design of the NEOTEM study. This figure represents the design of the current clinical trial involving 35 postsurgery patients. It compares the 2 treatment regimens: the intervention group receives neoadjuvant temozolomide followed by chemoradiotherapy (CRT) and then adjuvant temozolomide, while the control group undergoes CRT followed by adjuvant temozolomide. The figure details the cycles and durations of each treatment phase for both groups.

Patients in the control arm received CRT followed by 6 cycles of adjuvant TMZ starting at least 4 weeks after completing RT. The TMZ dose and schedule, as well as the RT prescription, were the same as in the intervention arm (Figure 1).

### Surveillance and Follow-Up

Patients were visited weekly during RT and monthly during neoadjuvant and adjuvant chemotherapy by radiation oncology specialists. If clinical progression was suspected in the intervention arm before the completion of the 3 cycles of neoadjuvant TMZ, patients immediately started the CRT treatment.

Bloodwork tests were carried out periodically 24–72 h before receiving the next cycle of TMZ and weekly during the CRT. Mini-Mental State Examination (MMSE) questionnaires were performed at different time points to test the cognitive function: (a) before starting the nTMZ, (b) before and immediately after CRT, and (c) after completing the adjuvant TMZ in the intervention group.^22^ In the control group, MMSE was performed before and immediately after CRT and after completing the adjuvant TMZ.

In both groups, multiparametric MRI with Magnetic Resonance Spectroscopy (MRS) and Magnetic Resonance Perfusion (MRP) sequences were done before starting the treatment, which was 2–4 weeks after surgery. In the intervention group, the MRI was repeated 2–4 weeks after the completion of 3 cycles of nTMZ or at the time of neurological deterioration in case tumor progression was suspected. The clinical progression was defined as any deterioration in mental and cognitive function, the emergence of focal neurological deficit, seizure, and headache that were attributable to a brain tumor. Another sign of progression was the start of dexamethasone to control the symptoms. In both groups, patients were followed by MRI 12 weeks after the completion of the CRT and then every 3–6 months for 2 years. If neurological symptoms worsened, the brain MRI was done earlier. The follow-up period was planned to be at least 24 months.

### Molecular Studies

Molecular studies were not mandatory but strongly encouraged. *IDH* was tested by IHC (immunohistochemistry) or genetic studies, including sequencing analyses to identify mutations in *IDH1*, *IDH2*, and *TERT* genes. O^6^-methylguanine-DNA methyltransferase status was defined as the presence of methylation in the *MGMT* gene using pyrosequencing.^23^

### Statistical Analysis

The primary endpoint of this trial was the PFS, defined as the time without disease progression from the randomization to the time of death or last follow-up or clinical or radiological progression. Secondary endpoints included OS, radiological and clinical response to nTMZ, and safety measured by the rate of adverse events. Radiological response to nTMZ was defined based on RECIST 1.1 criteria by comparing MRI sequences at baseline (pre-nTMZ) and after the administration of 3 cycles of nTMZ (post-nTMZ) in the intervention arm. The toxicities were graded based on the Common Terminology Criteria for Adverse Events version 5.0 (CTCEA v5.0). The calculated sample size with an 80% power (2-sided α of 0.05) was 67 patients in each group (134 patients in total), aiming to detect a hazard ratio (HR) of 0.6 or less, meaning at least a 40% relative reduction in disease progression in 6 months. About one-third of the patients were subjected to an interim analysis to assess the futility of the intervention. An intention-to-treat (ITT) analysis was performed to include all randomized patients in the analysis according to the group they were originally assigned to, regardless of whether they completed the intervention as per protocol. The Cox proportional hazards test method was used to compare the PFS between the 2 groups and the effect of different variables, including ECOG, the extent of resection, and MGMT methylation status. ANOVA was used for repeated measures to compare changes in cognitive status scores. The level of significance was placed at 0.05.

## Results

### Study Participants

**Figure 2.**
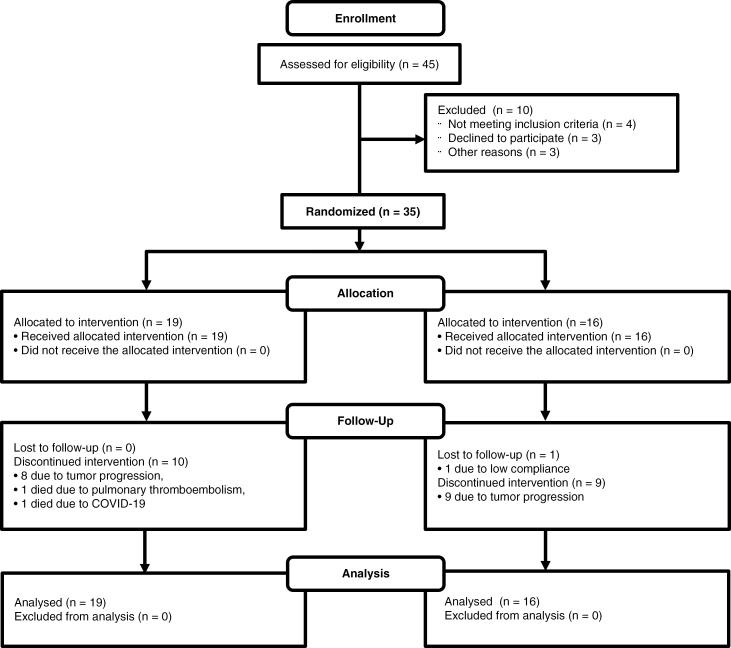
The CONSORT diagram of the NEOTEM study. CONSORT diagram of the NEOTEM trial indicates that out of 45 patients assessed for eligibility, 35 were randomized. The intervention group had 19 patients, all of whom received the allocated intervention. During follow-up, 10 patients discontinued the intervention (8 due to tumor progression, 1 due to pulmonary thromboembolism, and 1 due to COVID-19). The standard treatment group included 16 patients, all of whom received the allocated intervention, with 9 discontinuing due to tumor progression and 1 lost to follow-up due to low compliance. All patients in both groups were included in the final analysis.

Due to the slow recruitment of patients, which was coincident with the COVID-19 epidemic, only 35 patients were randomized during the study period: 19 patients to the control group and 16 patients to the intervention group (Figure 2). An interim futility analysis was carried out. Table 1 summarizes the patient and tumor characteristics.

**Table 1.** Patients and Tumor Characteristics

Characteristics	Intervention (*n* = 16)	Control (*n* = 19)	*P*-value
Age, average	56.6 y (±19)	52.4 y (±14.9)	0.344
Sex			
Male	11 (68.8%)	13 (68.4%)	
Female	5 (31.2%)	6 (31.6%)	
ECOG			1.000
0–1	9 (56.3%)	10 (52.6%)	
2–3	7 (43.8%)	9 (47.4%)	
MMSE score, average	18.1 (±9.1)	23.6 (±5.7)	0.054
Extent of resection			0.404
Biopsy	8 (50%)	7 (36.8%)	
STR	5 (31.6%)	6 (31.6%)	
NTR	2 (12.5%)	6 (31.6%)	
GTR	1 (6.3%)	0	
Resectable			0.506
Yes	8 (50%)	12 (63.2%)	
No	8 (50%)	7 (36.8%)	
Tumor Extension			0.018
Unifocal	4 (25%)	13 (68.4%)	
Multifocal	12 (75%)	6 (31.6%)	
Laterality of tumor			0.379
Unilateral	12 (75%)	29 (89.5%)	
Bilateral	4 (25%)	2 (10.5%)	
MGMT^a^			0.695
Methylated	8 (57.1%)	6 (42.9%)	
Non-methylated	5 (41.7%)	7 (58.3%)	
*IDH* mutation^a^			1
Yes	1 (11.1%)	3 (21.4%)	
No	8 (88.9%)	11 (78.6%)	

Abbreviations: ECOG, Eastern Cooperative Oncology Group; GTR, gross tumor resection; IDH, isocitrate dehydrogenase; MGMT, O^6^-Methylguanine-DNA Methyltransferase; MMSE, Mini-Mental State Examination; NTR, near-total resection; STR, sub-total resection.

^a^Out of available cases with molecular testing.

### Treatments and Outcomes

Treatments outcomes are summarized in Supplementary Table 1. The number of neoadjuvant chemotherapy cycles given in the intervention group was as follows: 11 (68.8%) patients received 3 cycles of TMZ, 2 (12.5%) patients received 2 cycles due to early disease progression and commencement of RT and death in one patient, 3 (18.8%) patients received only 1 cycle of TMZ due to early disease progression: 1 patient was lost to follow-up, another progressed and died, and the third commenced RT earlier due to disease progression. Of the 16 patients in the intervention arm, 9 (56.2%) underwent CRT with 60 Gy in 30 fractions, while 3 (18.7%) received hypofractionated RT with 40 Gy in 15 fractions due to the deterioration of PS after progression on nTMZ. The remaining 4 patients in the intervention arm did not proceed with CRT for the following reasons: 3 had a baseline ECOG of 3 and experienced rapid clinical deterioration, with no improvement following chemotherapy. This decline in PS made them ineligible for radiotherapy. The fourth patient, despite having an ECOG of 1, chose to discontinue treatment and refused radiotherapy. Post-CRT, 7 (43.7%) patients completed 3 cycles of adjuvant TMZ. Two (12.5%) patients finished 2 cycles, and one (6.2%) patient received only 1 cycle. Three (18.7%) patients did not receive any adjuvant TMZ due to poor PS, tumor progression, or death.

In the control group, 15 (78.9%) patients underwent CRT with 60 Gy in 30 fractions plus TMZ. However, 4 (21%) patients died during RT: one due to COVID-19 infection, one due to pulmonary thromboembolism, and 2 due to tumor progression. Regarding TMZ administration, 9 (47.3%) patients completed more than 6 cycles. Two (10.5%) patients received 5 cycles, another 2 (10.5%) received 2 cycles, and 1 (5.2%) patient completed only 1 cycle. The adjustments in the number of TMZ cycles were due to factors such as tumor progression, low PS, or death.

**Figure 3.**
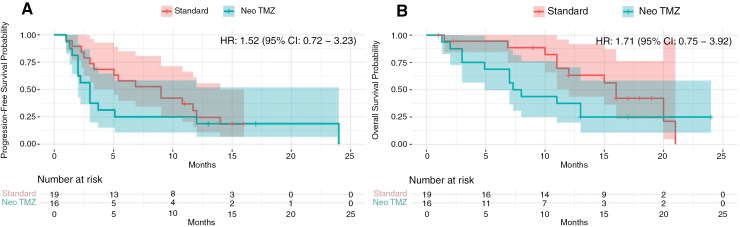
Progression-free survival (A) and overall survival (B) in the intervention (Neo TMZ) versus control (Standard) groups. The Kaplan-Meier curves illustrate the progression-free survival (PFS) and overall survival (OS) probabilities over time for patients in the intervention group (Neo TMZ) compared to those in the control group (Standard). (A) Median PFS was 3 months (95% CI, 1.98–4.01) in the intervention group versus 9 months (95% CI, 3.93–14.06) in the control group, with a hazard ratio (HR) of 1.52 (95% CI, 0.72–3.23). (B) Median OS was 7.3 months (95% CI, 5.34–9.26) in the intervention group versus 16 months (95% CI, 14.22–17.78) in the control group, with an HR of 1.71 (95% CI, 0.75–3.92; *P* = .198). The number at risk at different time points is shown below each plot.

After a median follow-up of 13 months, there was no PFS or OS benefit in the intervention group. The median PFS in the intervention versus control group was 3 months (95% confidence interval [Cl]: 1.98–4.01) versus 9 (95% Cl: 3.93–14.06) months (HR: 1.52, [CI 95% = 0.72–3.23], *P* = .737). The 6-month PFS rates were 25% versus 58% (*P* = .042), and 12-month PFS rates were 25% versus 26% (*P* = .390) in the intervention and control arms, respectively. Median OS in the intervention versus control groups was 7.3 (95% CI: 5.34–9.26) versus 16 (95% CI: 14.22–17.78) months (HR: 1.71 [95% CI: 0.75–3.92], *P* = .198). The 6-month OS rates were 69% versus 94% (*P* = .062), and 12-month OS rates were 38% versus 70% (*P* = .057) in the intervention and control arms, respectively. Figure 3 shows PFS and OS rates in both study arms.

Tumor progression observed during the treatment with nTMZ was high, reaching 73.4%, with only 2 patients (13.3%) experiencing a partial response and another 2 patients (13.3%) achieving radiological stability. Patients who had a limited extent of resection (biopsy or STR) had a higher progression rate (84.6%) compared to those who had a larger extent (NTR or GTR) of resection (84.6% versus 0%, *P* = .057). However, even though the difference was marginally nonsignificant, it suggests a trend where larger extents of resection may be associated with lower progression rates. Also the median PFS was 12 months (95% CI: 6.64–17.85) in patients with progression and 2 months (95% CI: 1.73–3.14) in patients with stable or partial response, respectively (*P* = .034). Due to the small sample size, these results should be interpreted cautiously, and further studies with larger samples are recommended to confirm these findings.

### Subgroup Analysis

The median OS and PFS in the m-MGMT and um-MGMT subgroups are shown in Supplementary Figure 1A, B. The median PFS in the subgroup of patients with m-MGMT was 3.7 months (95% CI: 1.90–5.49) and 3 (95% CI: 1.92–4.07) in the intervention and control groups, respectively (*P* = .230). The median PFS in the subgroup of patients with um-MGMT was 2.2 (95% CI: 1.68–2.71) and 10.8 (95% CI: 6.18–15.41) months in the intervention and control groups, respectively (*P* < .001) (Supplementary Figure 1B).

The median OS and PFS in the high PS (ECOG 0–1) and low PS (ECOG 2–3) subgroups is shown in Supplementary Figure 1C, D. The median PFS in the subgroup of patients with high PS (ECOG 0–1) was 3.7 (95% CI: 0.72–4.98) versus 10.8 (95% CI: 7.32–14.85) in the intervention and control group, respectively (*P* = .032). The median PFS in the subgroup of patients with low PS (ECOG 2-3) was 2 (95% CI: 0.78–4.72) versus 3 (95% CI: 1.2–5.21) months in the intervention and control groups, respectively (*P* = .23) (Supplementary Figure 1D).

The median OS and PFS in the limited (biopsy and STR) and larger (NTR, GTR) extent of resection subgroups is shown in Supplementary Figure 1E, F. The median PFS in the subgroup of patients with limited surgical resection (biopsy or STR) was 2.4 (95% CI: 1.32–4.85) versus 5.5 (95% CI: 2.72–7.98) in the intervention and control groups, respectively (*P* = .27). The median PFS in the subgroup of patients with NTR and GTR was 24 (95% CI: 15.96–31.18) versus 8 (95% CI: 4.61–13.08) months in the intervention and control group, respectively (*P* = .039) (Supplementary Figure 1F).

The rate of adverse events was not statistically different between the 2 arms (summarized in Supplementary Table 2). No grade 5 toxicity was seen in the intervention group. There was one case of grade 4 thrombocytopenia, which was managed by permanent discontinuation of TMZ, and 2 cases of grade 3 toxicity in the form of treatment-related thrombocytopenia and neutropenia during the neoadjuvant course, leading to 75% TMZ dose reduction for the subsequent cycle. In the control group, 1 patient presented with sepsis and consequently grade 5 cytopenia, 1 patient had grade 3 thrombocytopenia, and 1 patient experienced grade 3 pulmonary embolism after the first course of adjuvant TMZ, and after the second course, the same patient presented with grade 3 thrombocytopenia.

In the intervention group, no nonhematological complications were recorded. In the control group, two patients had nonhematological complications (1 patient was hospitalized due to neurological decline on the 27th day of CRT in the context of septic shock, and one case had pulmonary embolism) (*P* = .489).

## Discussion

This phase II randomized controlled trial investigated the efficacy and safety of adding nTMZ to standard CRT treatment in patients with newly diagnosed GBM. We found that nTMZ did not significantly improve the PFS compared to the standard treatment regimen. The median PFS was 3 months in the intervention group and 9 months in the control group (*P* = .737). Importantly, a high proportion of patients (73.4%) experienced disease progression during nTMZ treatment. Adverse events were comparable between the 2 study arms. Acknowledging that the study was closed early due to futility is essential. While the study did not demonstrate a statistically significant detriment to median PFS with the addition of nTMZ, the trends observed, particularly the higher progression rates in the intervention group, are concerning. With a small sample size and early study closure due to futility, these trends raise questions about the potential negative impact of nTMZ on disease control. Thus, based on the current data, the results do not support the use of nTMZ in this patient population.

Despite these overall results, subgroup analyses yielded some noteworthy insights. For example, patients who underwent limited resections had higher progression rates (84.6%) compared to those who had more extensive resections (0%). The difference is close to statistical significance (*P* = .057), suggesting a potential trend favoring more extensive resections for lower progression rates. Moreover, the subgroup analysis showed a lower median PFS and a significant detrimental effect of nTMZ in patients with um-MGMT and good PS. Additionally, patients who underwent NTR or GTR had better outcomes with nTMZ, which needs future validation. It is well known that biopsy-only patients have a worse prognosis and may present with worse PFS.^24^ Most patients in the present study (43%) underwent biopsy only. This may represent an important confounding factor when evaluating the efficacy of nTMZ. The reason for the high numbers of biopsies and debulking surgery instead of GTR or NTR is the conception of neurosurgeons regarding the incurable nature of GBM that prohibits them from doing more extended and riskier resections in addition to the location of the tumor in eloquent areas, specifically in the 74% of patients (those who underwent biopsy or STR), and lack of updated devices that ensure a safer maximal resection.^25^ While our findings provide insight into the differences in PFS among various subgroups, the small sample size limits the statistical power of these analyses. As such, these results should be interpreted cautiously, and further studies with larger cohorts are necessary to confirm these observations.

Table 2 provides a summary of existing clinical trials assessing novel therapeutic approaches in newly diagnosed GBM.^4,7,8,26^ Compared to large-scale studies that established the current SOC treatment for newly diagnosed GBM, that is the Stupp regimen, our results explore a novel approach of administering TMZ for 3 cycles before the CRT (60 Gy in 30 sessions of 2 Gy/F, 5 days a week plus 75 mg/m^2^/d daily TMZ concurrently with RT) and thus a direct comparison in terms of efficacy is not applicable. However, our findings on PFS fall within the range observed in these more extensive studies (typically 6–12 months) for the standard treatment. This suggests that while nTMZ did not result in PFS improvement, it also did not significantly worsen outcomes compared to the established approaches. The strength of our work lies in exploring a potential new avenue for treatment personalization, particularly for patients with m-MGMT and those with NTR or GTR, warranting further investigation.

**Table 2.** Summary of Clinical Trials Exploring Novel Therapeutic Approaches in Newly Diagnosed GBM

Authors	Study type	No. of samples	Interventio*n* group	Control group	Studied outcome	Results	Conclusion
CENTRIC EORTC, 2014^26^	Phase III	545	Cilengitide 2000 mg of the drug intravenously twice weekly	Standard Stupp Protocol	OS	Median OS = 26.3 m versus 26.3 m	The addition of cilengitide did not provide any survival benefit
EF-14 trial, 2017^4^	Phase III	695	Receive TTFields plus maintenance temozolomide chemotherapy	Standard Stupp Protocol	OS and PFS	Median OS = 20.9 m versus 16 m. Median PFS = 6.7 m versus 4 m	TTFields are a valuable addition to the standard maintenance therapy
CeTeG/NOA–09, 2019^8^	Phase III	141 methylated MGMT	6 courses of lomustine (100 mg/m^2^ on day 1) plus TMZ (100–200 mg/m^2^/day on days 2–6 of the 6-week course) in addition to RT (59–60 Gy)	Standard Stupp Protocol	OS	Median OS was 48.1 versus 31.4 months (*P* = ·0492)	Lomustine-plus TMZ might improve survival compared with TMZ with methylated MGMT promoter
CheckMate-498, 2023^7^	Phase III	498 unmethylated MGMT	Nivolumab 240 mg every 2 weeks for 8 cycles, then 480 mg every 4 weeks	Standard Stupp Protocol	OS, PFS, TRAE	Median OS = 13.4 m versus 14.9 m (*P* = .0037). Median PFS = 6 m versus 6.2 m TRAE = 21.9% versus 25.1%	TMZ + RT demonstrated a longer mOS than NIVO + RT
Current study NEOTEM	Phase ll	35	Max. 3 cycles of TMZ 150–200 mg/m^2^/day for 5 days before RT	Standard Stupp Protocol	PFS and RR	PFS = 9 m versus 3 m HR = 1.52 *P* = .737 OS = 16 m versus 7.3 m HR = 1.71 *P* = .198 (NS)	Overall, there was no benefit, but in NTR/GTR and m-MGMT, a benefit was observed

Abbreviations: GTR, gross tumor resection; MGMT, O^6^-methylguanine-DNA methyltransferase; m-MGMT, methylated-MGMT; NTR, near-total resection; OS, overall survival; PFS, progression-free survival; RR, response rate; RT, radiation therapy; TMZ, temozolomide; TRAE, treatment-related adverse event; TTFields, tumor-treating fields.

Despite the potential advantages of nTMZ, the present study failed to demonstrate any benefit in the established study endpoints. In fact, the median PFS (9 versus 3 months, *P* = .737) and OS (16 versus 7.3 months, *P* = .198) were higher in the control versus intervention group.

Some of the reasons that may explain these results include:

(a) Imaging during treatment and follow-up was done in a shorter interval in the intervention group. In the control group, patients were included on the waiting list for RT, which could last up to 2 months from the time the post-op brain MRI was obtained. Subsequently, they underwent a 6-week RT and were reassessed with a new brain MRI 3 months later. In summary, subclinical radiological progression could not be detected in this group of patients for about 6 to 7 months. In contrast, in the intervention group, patients were examined with MRI following nTMZ, which was about 2.5 months after their baseline MRI.
(b) Although the patients and tumor characteristics were well balanced between the 2 groups, there were significant differences in tumor extension, with the intervention group having a higher rate of multifocal tumors (75%) compared to the control group (31.6%) (*P* = .018). These differences in tumor extension, with significantly more multifocal tumors in the intervention group, could have influenced the PFS outcomes and might account for the observed differences in survival benefits between the groups.
(c) During the interval from surgery to RT in the neoadjuvant group, 73.4% of patients progressed. Due to these rapid progressions, hypofractionated RT was recommended as patients’ PS decreased. This situation raises concerns about the efficacy of neoadjuvant chemotherapy, particularly regarding its impact on the timing and tolerability of subsequent radiation therapy. While hypofractionated RT is often employed in elderly and low KPS patients, improving outcomes in these groups, they generally still exhibit worse outcomes than the younger population receiving the full Stupp regimen.^27,28^ Moreover, the addition of nTMZ did not result in a notable improvement in PS or disease control that would make these patients eligible for conventional radiotherapy. This underscores the challenges associated with delayed RT after neoadjuvant chemotherapy, as disease progression during this period can significantly limit treatment options. Future studies should focus on strategies to optimize treatment timing and mitigate disease progression to enhance outcomes in this challenging patient subset.
(d) Temozolomide monotherapy seems not to be a sufficient option for obtaining optimal tumor control. Given the aggressive nature of GBM, achieving effective management typically requires a combination of different therapies. Perhaps more favorable results would have been achieved if neoadjuvant chemotherapy was started earlier (2–4 weeks following biopsy or STR). A large percentage of patients did not have sufficient resection before RT, which is a robust local treatment that improves local control if started earlier. This is the main confounding factor of our results. Subsequent studies should focus on patients with maximal safe surgical resections (NTR and GTR).
(e) Based on previous studies, neoadjuvant chemotherapy in the biopsied-only GBM patients showed inconclusive results. Moreover, it has been recommended to assess the effect of MGMT status on treatment response and survival.^29^ Therefore, better results could have been obtained if patient selection had been limited to those with m-MGMT and NTR or GTR.
(f) We opted to perform multiparametric MRI, including MRS and perfusion imaging, which can identify the recurrences more accurately than conventional MRI. This could, in part, contribute to a higher rate of progression diagnosis in the neoadjuvant arm.

Table 3 presents the findings of the main nTMZ trials in cases of GBM or high-grade glioma comprehensively.^16,30–35^ Furthermore, we will discuss 3 studies that have explored the role of nTMZ in GBM. Jiang and colleagues performed a retrospective study of 375 GBM patients from 2012 to 2018. A total of 163 patients were treated with TMZ 75 mg/m^2^ daily within 7 days of surgery until RT, for an average of 28 days. Subsequently, patients received CRT and adjuvant TMZ according to the Stupp protocol. A total of 220 patients underwent standard Stupp protocol exclusively. Both PFS and OS rates were significantly higher in the intervention group than in the controls. There was no difference in PFS and OS in those who underwent GTR, while in those who underwent less than a GTR (ie, biopsy, STR, or NTR), there was a significant difference in PFS and OS in favor of the neoadjuvant group (*P* = .0094 for PFS and *P* = .0004 for OS). In the m-MGMT subgroup, the difference in PFS and OS was statistically significant (*P* = .012 for PFS and *P* < .0001 for OS), while in the um-MGMT group, it was not.^35^

**Table 3.** Description of the Main Neoadjuvant TMZ Trials

Authors	Study type and sample size, histology	Design	Studied outcome	Results	Conclusion
Gilbert et al., 2002^30^	Single arm, 36, HGG	Max. 4 cycles of TMZ 200 mg/m^2^/day for 5 days before RT	RR, OS, PFS, and evaluated by MRI	11% CR and 31% PR, 28% SD, and 26% no response OS = 13.2 and PFS = 3.9 m	Safe and well-tolerated
Barada et al., 2005^31^	Single arm, 126, HGG	TMZ (200 mg/m^2^/day) on days 1–5 for 2 cycles	RR evaluated by MRI	20% (*n* = 32) partial response, 44% (*n* = 72) stable disease, 36% (*n* = 22) progression, OS was 16 in responders versus 3 m in patients with progressed disease	Primary chemotherapy is feasible
Chinot et al., 2007^32^	Single arm, 29, GBM	TMZ (150 mg/m^2^/day) on days 1–7 and 15–21 every 28 days (7 days on/7 days off) before RT	RR, OS, and PFS, evaluated by MRI	24% had a partial response, 31% were stable, and 41% had progressive disease, with median PFS = 3.8 m and median OS = 6.1 m. High response rate (55%), PFS (5.5 m), and OS (16 m) in patients with MGMT methylation	Modest efficacy of neoadjuvant dose-dense TMZ, inferior to standard concomitant chemoradiotherapy in the whole group of patients
Lou et al., 2013^33^	Single arm, 41, GBM	TMZ (200 mg/m^2^/day) on days 1–5 for 4 cycles, BEV (10 mg/kg) on days 1 and 15	RR evaluated by MRI, toxicity	24.4% partial response, 68.3% stable disease, 2.4% progression	Should be investigated in phase III trials
Ying Mao et al., 2015^34^	Phase II clinical trial, 99, GBM	TMZ 75 mg/m^2^/day for 2 weeks before RT versus Stupp Protocol	OS, PFS, toxicity	Median OS = 17.6 m, PFS = 13.2 *P* < .05, PFS nonsignificant	Favorable long-term survival
Shenouda et al., 2017^16^	Phase II clinical trial, 50, GBM	TMZ 75 mg/m^2^/day for 2 weeks before RT	OS, PFS, toxicity	Median OS = 22.3 m, PFS = 13.7 m, 4-year OS = 30.4%	Favorable long-term survival
Jiang et al., 2019^35^	Retrospective, 375 (163 NEO, 212 ADJ), GBM	Super-early initiation of TMZ within 7 days after craniotomy before RT versus Stupp Protocol	OS, PFS	OS = 23 m versus 17 m HR = 0.583, 95% CI 0.384–0.884, *P* = .011 PFS = 11.5 versus 9 m (NS)	May confer to survival benefits, especially for those without GTR or methylated MGMT
Current study NEOTEM	Phase II RCT, 35, GBM	TMZ (200 mg/m^2^/day) on days 1–5 for 3 cycles before RT versus Stupp Protocol	PFS, RR evaluated by MRI	13.3% Partial response, 13.3% stable disease, 73.4% progression	No overall benefit but in NTR/GTR and m-MGMT, a benefit was observed

Abbreviations: BEV, bevacizumab; CR, complete response; GTR, gross tumor resection; GBM, glioblastoma multiforme; HGG, high-grade glioma; MGMT, O^6^-Methylguanine-DNA Methyltransferase; NTR, near-total resection; OS, overall survival; PFS, progression-free survival; PR, partial response; RR, response rate; TRAE, Treatment-Related Adverse Event; TMZ, temozolomide.

In a nonrandomized single-arm phase II trial, Shenouda et al., studied 50 GBM patients who were treated with nTMZ 75 mg/m^2^ with an interval of 2–3 weeks after surgery for 2 weeks followed by hypofractionated CRT delivering 60 Gy in 20 fractions followed by 6 cycles of adjuvant TMZ. The median follow-up was 44 months. The median OS and PFS rates were 22.3 (95% CI: 14.6–42.7) and 13.7 (95% CI: 8.0–33.3) months, respectively. This study concluded that nTMZ provided a survival benefit with acceptable side effects. Moreover, median OS and PFS in m-MGMT patients (53.8 and 19.6) were significantly higher than in the um-MGMT group (16.2 and 8.5) (*P* = .01). Median OS was significantly higher in patients who underwent gross total or partial resection compared to those who underwent biopsy-only (24.2 versus 8.8, *P* = .001). However, the analysis did not indicate a statistically significant difference in median PFS between the 2 groups (20.3 versus 10.8, *P* = .47).^16^

It is worth noting that in both Jiang and Shenouda’s studies, the neoadjuvant TMZ treatment duration was shorter than our approach. This difference in the timing of treatment may have contributed to the differing observed outcomes. However, we tried to design our neoadjuvant TMZ more similarly to other solid tumors, such as rectal cancer, where the longer durations of neoadjuvant treatment, like total neoadjuvant therapy, have been shown to be effective and practice-changing.^36^

A study conducted by Malmström and colleagues investigated the effects of administering nTMZ before RT in patients with anaplastic astrocytoma (AA) and GBM. The study took place between 2003 and 2014 and enrolled 144 patients. Half of the patients were treated with TMZ before RT (receiving 200 mg/m^2^ of TMZ on days 1–5 every 28 days, followed by 60 Gy of radiotherapy in 30 fractions), concurrent TMZ was also administered daily with RT. The other half of the patients received CRT after surgery according to the SOC. The median survival was 20.3 months for CRT alone and 17.7 months for nTMZ, which did not reach the primary objective of the study. However, in a preplanned subgroup analysis, patients with AA treated with nTMZ showed a significant increase in median survival of 95.1 months compared to 35.2 months for the group receiving RT alone. For patients with GBM, there was no significant difference in survival.^37^

Our findings on nTMZ align with previous studies exploring neoadjuvant treatments for GBM. Similar to our research, these studies have not shown a significant PFS benefit with neoadjuvant approaches. However, our work adds to the growing body of evidence suggesting that potential subgroups, including patients with m-MGMT and NTR, or GTR might respond better. The MGMT methylation analysis aligns with prior research exploring the role of molecular markers in personalizing neoadjuvant therapy for GBM patients. Further investigation into these subgroups and potentially incorporating other molecular markers is needed to refine neoadjuvant treatment strategies for newly diagnosed GBM.

The present study has strengths and limitations that need to be acknowledged. One of the strengths includes its randomized design, which remains the gold standard for evaluating the efficacy and safety of any intervention. Randomized assignment of patients to the intervention (nTMZ) or control group (SOC) allows for minimizing the potential bias, yielding more reliable results. Another advantage was the enrollment of GBM patients who were treated in 1 center with a uniform treatment protocol. We also used multiparametric MRI with MRS and perfusion MRI in addition to the conventional sequences. MRS helps to differentiate between tumor recurrence and pseudoprogression, providing a more precise diagnosis.^38^ Additionally, combining perfusion parameters with standard MRI sequences improves the characterization of high-risk areas, ensuring that high-dose treatment volumes are accurately targeted based on both metabolic and perfusion characteristics.^39^ Another strength was the investigation of nTMZ, which addresses a novel approach to GBM treatment by investigating the potential benefits of adding TMZ, including shrinking the tumor before standard CRT and allowing for a more precise radiation treatment planning.

In addition, the subgroup analysis explored outcomes based on MGMT methylation status that provide valuable insights into treatment personalization for GBM patients. Finally, the evaluation of safety by assessing the rate of adverse events associated with the administration of nTMZ helps to determine its tolerability.

On the other hand, one of the main limitations of this study is its small sample size. With 35 patients enrolled, the study might be underpowered to detect statistically significant differences in PFS, particularly in the subgroup analysis. A larger sample size would have strengthened the generalizability of the findings. Another noticeable limitation was the high rate of progression during nTMZ. The proportion of patients experiencing disease progression during nTMZ raises questions about its effectiveness and the optimal duration of this therapy. A possible solution would be stratification at the time of recruitment, if done according to the potential confounding factors, and should be considered in subsequent studies. Another suggestion is to exclude patients with unresectable tumors (biopsied-only), as progression without a prompt CRT start is highly likely. It should also be noted that while the study explored the status of MGMT methylation, the investigation of additional molecular markers could provide a more comprehensive picture of potential responders to nTMZ. Moreover, the study follow-up period was relatively short, with a median of 13 months, which might be limiting for capturing long-term effects on OS. A more extended follow-up period would provide more robust data on OS.

## Conclusion

In this phase II randomized controlled trial, while nTMZ was well-tolerated with no increase in adverse events, it did not demonstrate a significant improvement in PFS compared to the standard treatment approach in the interim analysis. The median PFS was shorter in the intervention group (3 months) compared to the control group (9 months), although no significant difference was observed between the groups. A high proportion of patients (73.4%) receiving nTMZ experienced disease progression during this phase.

The potential benefit for patients with m-MGMT and NTR, or GTR who received neoadjuvant TMZ was observed, as they exhibited a longer median PFS than those with um-MGMT and those who underwent biopsy or STR. Thus, these findings suggest a possible role for these factors in guiding treatment decisions. However, due to the small sample size, drawing definitive conclusions, especially regarding the impact of the extent of resection on outcomes and subgroup analysis, is challenging. Therefore, future studies with larger sample sizes and stratification of patients according to confounding factors, such as the extent of surgical resection and more extended follow-up periods, are necessary to definitively determine the role of nTMZ in GBM treatment for selected patients, particularly those with specific molecular profiles. This approach may be beneficial only for specific subgroups of GBM patients, which need to be identified further to develop personalized treatment strategies.

## Supplementary Material

vdae195_suppl_Supplementary_Tables_S1-S2_Figure_S1

## Data Availability

The data generated and analyzed during the current study is not publicly available, but it can be available from the corresponding author upon reasonable request. Requests for access to the dataset should be directed to the corresponding author. Any data shared will be de-identified to protect participant confidentiality.

## References

[1] Kotliarova S, Fine HA. SnapShot: glioblastoma multiforme. Cancer Cell. 2012;21(5):710–710.e1. 22624719 10.1016/j.ccr.2012.04.031

[2] Wen PY, Kesari S. Malignant gliomas in adults. N Engl J Med. 2008;359(5):492–507. 18669428 10.1056/NEJMra0708126

[3] Stupp R, Mason WP, Van Den Bent MJ, et al; European Organisation for Research and Treatment of Cancer Brain Tumor and Radiotherapy Groups. Radiotherapy plus concomitant and adjuvant temozolomide for glioblastoma. N Engl J Med. 2005;352(10):987–996. 15758009 10.1056/NEJMoa043330

[4] Stupp R, Taillibert S, Kanner A, et al Effect of tumor-treating fields plus maintenance temozolomide vs maintenance temozolomide alone on survival in patients with glioblastoma: a randomized clinical trial. JAMA. 2017;318(23):2306–2316. 29260225 10.1001/jama.2017.18718 PMC5820703

[5] Krigers A, Pinggera D, Demetz M, et al The routine application of tumor-treating fields in the treatment of glioblastoma WHO° IV. Front Neurol. 2022;13:900377. 35785334 10.3389/fneur.2022.900377 PMC9243748

[6] Lassman AB, Joanta-Gomez AE, Pan PC, Wick W. Current usage of tumor treating fields for glioblastoma. Neurooncol Adv. 2020;2(1):vdaa069. 32666048 10.1093/noajnl/vdaa069 PMC7345837

[7] Omuro A, Brandes AA, Carpentier AF, et al Radiotherapy combined with nivolumab or temozolomide for newly diagnosed glioblastoma with unmethylated MGMT promoter: an international randomized phase III trial. Neuro-Oncol. 2023;25(1):123–134. 35419607 10.1093/neuonc/noac099 PMC9825306

[8] Herrlinger U, Tzaridis T, Mack F, et al Lomustine-temozolomide combination therapy versus standard temozolomide therapy in patients with newly diagnosed glioblastoma with methylated MGMT promoter (CeTeG/NOA–09): a randomised, open-label, phase 3 trial. The Lancet. 2019;393(10172):678–688. 10.1016/S0140-6736(18)31791-4 30782343

[9] Das S, Sahgal A, Perry JR. Commentary: lomustine-temozolomide combination therapy versus standard temozolomide therapy in patients with newly diagnosed glioblastoma with methylated MGMT promoter (CeTeG/NOA-09): a randomised, open-label, phase 3 trial. Front Oncol. 2020;10:66. 32083011 10.3389/fonc.2020.00066 PMC7005933

[10] Friedman HS, Kerby T, Calvert H. Temozolomide and treatment of malignant glioma. Clin Cancer Res. 2000;6(7):2585–2597. 10914698

[11] Zhang K, Wang X-q, Zhou B, Zhang L. The prognostic value of MGMT promoter methylation in Glioblastoma multiforme: a meta-analysis. Fam Cancer. 2013;12(3):449–458. 23397067 10.1007/s10689-013-9607-1

[12] Szylberg M, Sokal P, Śledzińska P, et al MGMT promoter methylation as a prognostic factor in primary glioblastoma: a single-institution observational study. Biomedicines. 2022;10(8):2030. 36009577 10.3390/biomedicines10082030 PMC9405779

[13] Jo J, Williams B, Smolkin M, et al Effect of neoadjuvant temozolomide upon volume reduction and resection of diffuse low-grade glioma. J Neurooncol. 2014;120(1):155–161. 25038848 10.1007/s11060-014-1538-7

[14] Oshiro S, Tsugu H, Komatsu F, et al Efficacy of temozolomide treatment in patients with high-grade glioma. Anticancer Res. 2009;29(3):911–917. 19414327

[15] Franceschi E, Omuro AM, Lassman AB, et al Salvage temozolomide for prior temozolomide responders. Cancer. 2005;104(11):2473–2476. 16270316 10.1002/cncr.21564

[16] Shenouda G, Souhami L, Petrecca K, et al A phase 2 trial of neoadjuvant temozolomide followed by hypofractionated accelerated radiation therapy with concurrent and adjuvant temozolomide for patients with glioblastoma. Int J Radiat Oncol Biol Phys. 2017;97(3):487–494. 28011051 10.1016/j.ijrobp.2016.11.006

[17] Buszek SM, Al Feghali KA, Elhalawani H, et al Optimal timing of radiotherapy following gross total or subtotal resection of glioblastoma: a real-world assessment using the National Cancer Database. Sci Rep. 2020;10(1):4926. 32188907 10.1038/s41598-020-61701-z PMC7080722

[18] Chevli N, Elhalawani H, Mohamed A, et al RTHP-03. prognostic impact of timing between surgery and Radiotherapy (Rt) in patients with Glioblastoma (GBM). Neuro-Oncol. 2017;19(Suppl 6):vi219–vi219.

[19] Pollom EL, Fujimoto DK, Han SS, et al Newly diagnosed glioblastoma: adverse socioeconomic factors correlate with delay in radiotherapy initiation and worse overall survival. J Radiat Res. 2018;59(suppl_1):i11–i18. 29432548 10.1093/jrr/rrx103 PMC5868191

[20] Rykkje AM, Larsen VA, Skjøth-Rasmussen J, et al Timing of early postoperative MRI following primary glioblastoma surgery—A retrospective study of contrast enhancements in 311 patients. Diagnostics (Basel). 2023;13(4):795. 36832282 10.3390/diagnostics13040795 PMC9955136

[21] Niyazi M, Andratschke N, Bendszus M, et al ESTRO-EANO guideline on target delineation and radiotherapy details for glioblastoma. Radiother Oncol. 2023;184:109663. 37059335 10.1016/j.radonc.2023.109663

[22] Folstein MF, Folstein SE, McHugh PR. “Mini-mental state”: a practical method for grading the cognitive state of patients for the clinician. J Psychiatr Res. 1975;12(3):189–198. 1202204 10.1016/0022-3956(75)90026-6

[23] Weller M, van den Bent M, Preusser M, et al EANO guidelines on the diagnosis and treatment of diffuse gliomas of adulthood. Nat Rev Clin Oncol. 2021;18(3):170–186. 33293629 10.1038/s41571-020-00447-z PMC7904519

[24] Sales AH, Beck J, Schnell O, et al Surgical treatment of glioblastoma: state-of-the-art and future trends. J Clin Med. 2022;11(18):5354. 36143001 10.3390/jcm11185354 PMC9505564

[25] De Simone M, Conti V, Palermo G, De Maria L, Iaconetta G. Advancements in glioma care: focus on emerging neurosurgical techniques. Biomedicines. 2023;12(1):8. 38275370 10.3390/biomedicines12010008 PMC10813759

[26] Stupp R, Hegi ME, Gorlia T, et al; European Organisation for Research and Treatment of Cancer (EORTC). Cilengitide combined with standard treatment for patients with newly diagnosed glioblastoma with methylated MGMT promoter (CENTRIC EORTC 26071-22072 study): a multicentre, randomised, open-label, phase 3 trial. Lancet Oncol. 2014;15(10):1100–1108. 25163906 10.1016/S1470-2045(14)70379-1

[27] Roa W, Brasher P, Bauman G, et al Abbreviated course of radiation therapy in older patients with glioblastoma multiforme: a prospective randomized clinical trial. J Clin Oncol. 2004;22(9):1583–1588. 15051755 10.1200/JCO.2004.06.082

[28] Perry JR, Laperriere N, O’Callaghan CJ, et al; Trial Investigators. Short-course radiation plus temozolomide in elderly patients with glioblastoma. N Engl J Med. 2017;376(11):1027–1037. 28296618 10.1056/NEJMoa1611977

[29] Nabian N, Ghalehtaki R, Zeinalizadeh M, Balaña C, Jablonska PA. State of the neoadjuvant therapy for glioblastoma multiforme—Where do we stand? Neurooncol. Adv.. 2024;6(1):vdae028. 38560349 10.1093/noajnl/vdae028 PMC10981465

[30] Gilbert MR, Friedman HS, Kuttesch JF, et al A phase II study of temozolomide in patients with newly diagnosed supratentorial malignant glioma before radiation therapy. Neuro-Oncol. 2002;4(4):261–267. 12356356 10.1093/neuonc/4.4.261 PMC1920664

[31] Brada M, Ashley S, Dowe A, et al Neoadjuvant phase II multicentre study of new agents in patients with malignant glioma after minimal surgery. Report of a cohort of 187 patients treated with temozolomide. Ann Oncol. 2005;16(6):942–949. 15870090 10.1093/annonc/mdi183

[32] Chinot OL, Barrie M, Fuentes S, et al Correlation between O^6^-methylguanine-DNA methyltransferase and survival in inoperable newly diagnosed glioblastoma patients treated with neoadjuvant temozolomide. J Clin Oncol. 2007;25(12):1470–1475. 17442989 10.1200/JCO.2006.07.4807

[33] Lou E, Peters KB, Sumrall AL, et al Phase II trial of upfront bevacizumab and temozolomide for unresectable or multifocal glioblastoma. Cancer Med. 2013;2(2):185–195. 23634286 10.1002/cam4.58 PMC3639657

[34] Mao Y, Yao Y, Zhang L-W, et al Does early postsurgical temozolomide plus concomitant radiochemotherapy regimen have any benefit in newly-diagnosed glioblastoma patients? A multi-center, randomized, parallel, open-label, Phase II Clinical Trial. Chin Med J (Engl). 2015;128(20):2751–2758. 26481741 10.4103/0366-6999.167313 PMC4736883

[35] Jiang H, Zeng W, Ren X, et al Super-early initiation of temozolomide prolongs the survival of glioblastoma patients without gross-total resection: a retrospective cohort study. J Neurooncol. 2019;144(1):127–135. 31175579 10.1007/s11060-019-03211-1

[36] Ghalehtaki R, Nourbakhsh F, Abyaneh R, et al Optimal sequence for total neoadjuvant therapy in locally advanced rectal cancer: an evidence-based review. Cancer Medicine. 2024;13(19):e70291. 39387519 10.1002/cam4.70291 PMC11465286

[37] Malmström A, Poulsen HS, Grønberg BH, et al; Nordic Clinical Brain Tumor Study Group (NCBTSG). Postoperative neoadjuvant temozolomide before radiotherapy versus standard radiotherapy in patients 60 years or younger with anaplastic astrocytoma or glioblastoma: a randomized trial. Acta Oncol. 2017;56(12):1776–1785. 28675067 10.1080/0284186X.2017.1332780

[38] Zhang H, Ma L, Wang Q, et al Role of magnetic resonance spectroscopy for the differentiation of recurrent glioma from radiation necrosis: a systematic review and meta-analysis. Eur J Radiol. 2014;83(12):2181–2189. 25452098 10.1016/j.ejrad.2014.09.018

[39] Guo L, Wang G, Feng Y, et al Diffusion and perfusion weighted magnetic resonance imaging for tumor volume definition in radiotherapy of brain tumors. Radiat Oncol. 2016;11:1–13. 27655356 10.1186/s13014-016-0702-y PMC5031292

