# Supplementary material for: Postoperative NEOadjuvant TEMozolomide followed by chemoradiotherapy versus upfront chemoradiotherapy for glioblastoma multiforme (NEOTEM) trial: Interim results
Source: Neurooncol Adv. 2024 Nov 14;6(1):vdae195. doi: 10.1093/noajnl/vdae195 (PMC11632829; doi:10.1093/noajnl/vdae195)
Supplement: vdae195_suppl_Supplementary_Tables_S1-S2_Figure_S1 [file vdae195_suppl_Supplementary_Tables_S1-S2_Figure_S1.docx]

Table S1. Treatment outcomes in both study arms

| **Characteristics** | **Intervention**  **(n=16)** | **Control (n=19)** | **P-value** |
| --- | --- | --- | --- |
| **PFS**  **Median (months)**  **6 months (%)**  **12 months (%)** | 3 (CI= 1.98-4.01)  25% (Cl=0.03-0.47)  25% (Cl= 0.03-0.47) | 9 (CI= 3.93-14.06)  58% (Cl=0.36-0.80)  26% (Cl= 0.06-0.46) | 0.737  0.042  0.39 |
| **OS**  **Median (months)**  **6 months (%)**  **12 months (%)** | 7.3 (CI= 5.34-9.26)  69% (Cl= 0.45-0.93)  38% (Cl= 0.14-0.62) | 16 (CI= 14.22-17.78)  94% (Cl= 0.84-1.04)  70% (Cl= 0.48-0.96) | 0.198  0.062  0.057 |
| **Response to nTMZ**  **Partial response**  **Stable disease**  **Progression** | 2 (13.3%)  2 (13.3%)  11 (73.4%) | -  -  - |  |

PFS: Progression-Free Survival; OS: Overall Survival; nTMZ: neoadjuvant Temozolomide

Table S2. Adverse events are summarized and compared for the intervention and control groups.

| **Grade 3 or higher Toxicity** | **Intervention** | **Control** | **P value** |
| --- | --- | --- | --- |
| **Overall** | 3 (18.7) | 3 (15.8) | 1 |
| **Non-hematologic** | 0 | 2 (10.5) | 0.489 |
| **Hematologic** | 3 (18.7) | 3 (15.8) | 1 |

**
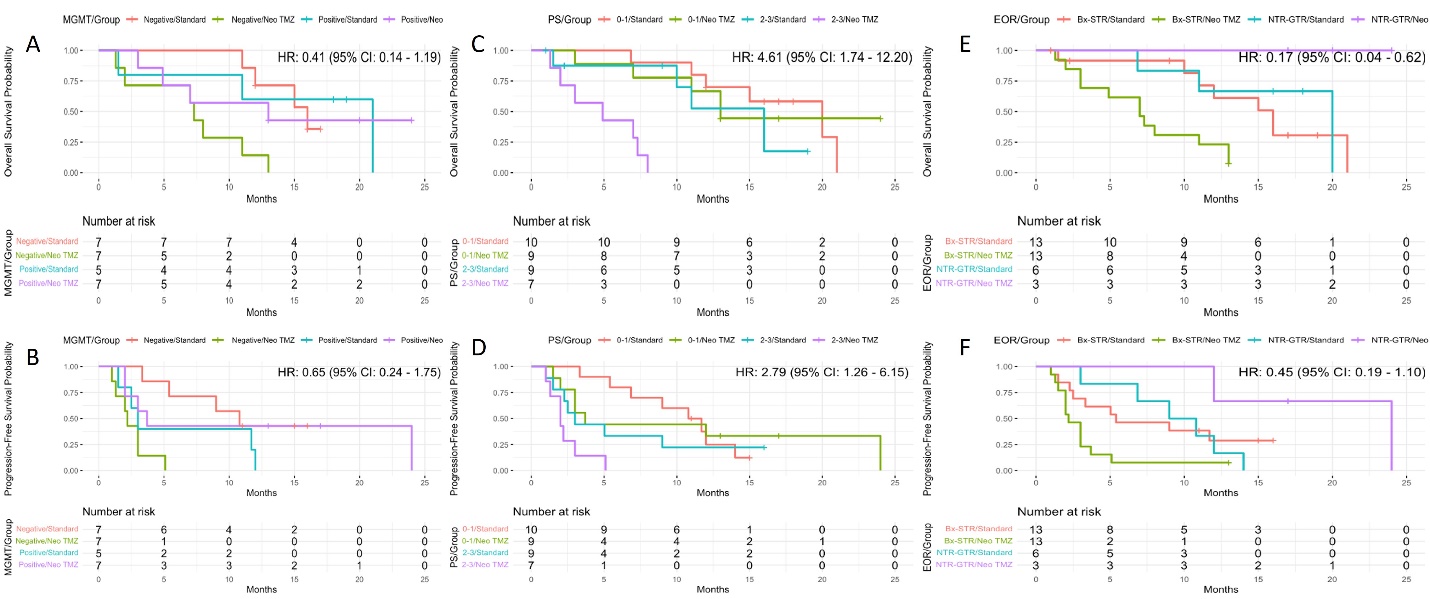
**

**Figure S1.** Progression-Free Survival (PFS) and Overall Survival (OS) in the intervention (Neo TMZ) versus control (Standard) groups based on MGMT status (A-B), performance status (PS) (C-D), and extent of resection (EOR) (E-F).

**Figure S1 Legend:**

A-B: Kaplan-Meier curves for overall survival (A) and progression-free survival (B) stratified by MGMT status (red line: Negative/Standard, green line: Negative/Neo TMZ, blue line: Positive/Standard, purple line: Positive/Neo TMZ). A: The hazard ratio (HR) for overall survival was 0.41 (95% CI, 0.14-1.19). B: The hazard ratio (HR) for progression-free survival was 0.65 (95% CI, 0.24-1.75).

C-D: Kaplan-Meier curves for overall survival (C) and progression-free survival (D) stratified by performance status (red line: 0-1/Standard, green line: 0-1/Neo TMZ, blue line: 2-3/Standard, purple line 2-3/Neo TMZ). C: The hazard ratio (HR) for overall survival was 4.61 (95% CI, 1.74-12.20). D: The hazard ratio (HR) for progression-free survival was 2.79 (95% CI, 1.26-6.15).

E-F: Kaplan-Meier curves for overall survival (E) and progression-free survival (F) stratified by extent of resection (red line: Bx-STR/Standard, green line: Bx-STR/Neo TMZ, blue line: NTR-GTR/Standard, purple line: NTR-GTR/Neo TMZ). E: The hazard ratio (HR) for overall survival was 0.17 (95% CI, 0.04-0.62). F: The hazard ratio (HR) for progression-free survival was 0.45 (95% CI, 0.19-1.10).

The number at risk at different time points is shown below each plot.
